# Reserve and Reserve-building activities research: key challenges and future directions

**DOI:** 10.1186/s12868-016-0297-0

**Published:** 2016-09-15

**Authors:** Carolyn E. Schwartz, Bruce D. Rapkin, Brian C. Healy

**Affiliations:** 1 DeltaQuest Foundation, Inc., 31 Mitchell Road, Concord, MA 01742 USA; 2 Departments of Medicine and Orthopaedic Surgery, Tufts University Medical School, Boston, MA USA; 3 Department of Epidemiology and Population Health, Division of Community Collaboration and Implementation Science, Albert Einstein College of Medicine, Bronx, NY USA; 4 Department of Neurology, Harvard Medical School, Boston, MA USA; 5 Partners MS Center at Brigham and Women’s Hospital, Boston, MA USA; 6 Massachusetts General Hospital Biostatistics Center, Boston, MA USA

**Keywords:** Reserve-building activities, Cognitive Reserve, Neurological Reserve, Resilience, Quality of life, Measurement, Nomenclature theory, Tipping point, Inter-disciplinary

## Abstract

**Background:**

The concept of Cognitive Reserve has great appeal and has led to an interesting and important body of research. We believe, however, that it is unnecessarily limited by ‘habits’ of measurement, nomenclature, and intra-disciplinary thinking.

**Main body:**

A broader, more comprehensive way of conceptualizing Reserve is proposed that invokes a broader measurement approach, nomenclature that uses specific terms embedded in a theoretical model, and crosses disciplines.

**Conclusion:**

Building on this comprehensive conceptualization, we will discuss fruitful directions for future research.

## Background

Research from many disciplines has attempted to characterize what makes some individuals more resilient to chronic illness and life stress. That is, given a stressful health or life event, some individuals experience less distress and damage than others. Nominated factors reflect disciplinary foci, ranging from sociodemographic resources [1]; psychological factors such as self-complexity [2] and self-efficacy [3]; behavioral factors such as problem-focused coping [4] and positive deviance [5]; and biological differences in stress response [6]. Recent work from the field of cognitive neuroscience has suggested that factors related to inborn resources and early-life enrichment can and do buffer individuals from the usual disability associated with impairment [7]. Building on the oft-noted disconnection between impairment and disability in neurological diseases, the *Cognitive Reserve hypothesis* posits that intellectual enrichment results in cognitive efficiency which provides a “Cognitive Reserve” against disease-related cognitive impairment [7–9]. Distinguished from inborn resources such as brain size or neuronal count—or “Brain Reserve” [7]—Cognitive Reserve is hypothesized to be an active process. Cognitive Reserve may involve either the mode in which tasks are handled due to more flexible or efficient brain networks; or compensatory systems reflecting the adoption of new brain networks [7]. Since originally described and discussed by Snowden [11] in 1996 and Stern in 2002 [12], research on Cognitive Reserve and Brain Reserve has burgeoned.

Recent research supports a protective effect of pre-morbid education level (a measure of Cognitive Reserve) [7] or head circumference (a measure of Brain Reserve) [10] against cognitive disability in a range of neurological conditions (e.g., multiple sclerosis (MS) [11, 12], Alzheimer’s disease [13], Parkinson’s disease [14]), as well as in regards to neurological or neuropsychological effects of toxic exposure (e.g., cancer chemotherapy [15], lead [16]) or trauma [17]. Higher pre-morbid education [18] has also been associated with structural magnetic resonance imaging (MRI) metrics of (lower) brain atrophy in MS patients [19] as well as functional MRI outcomes [20]. Smaller head circumference has been associated with lower scores on a cognitive screening test [21], increased risk of expressing dementia in late life [22], and a more rapid onset of progressive neurological disease [23]. A noradrenergic hypothesis has been suggested as a mechanism, such that neurocognitive correlates of noradrenergic activity—arousal, sustained attention, response to novelty, and awareness—mediate Cognitive Reserve’s protective effects [24, 25].

While the concepts of Cognitive Reserve and Brain Reserve have great appeal and have led to an interesting and important body of research, Reserve may be a broader construct rather than either Cognitive Reserve or Brain Reserve alone. Hence, a broader discussion of Reserve may be in order. We would like to suggest the following *axioms for conceptualizing Reserve*. First, it is multidimensional, including cognitive, physical, socio-emotional, and spiritual components. Second, it is an emergent phenomenon that is only observable with a referent. That is, one infers Reserve because an individual functions *better than expected* in the face of a physical or psychological loss of capacity. Third, while the imperfect correlation between physical status and performance suggests Reserve is present, Reserve can be exhausted or overwhelmed by disease burden. A precipitous drop in performance even without an obvious change in physical status would suggest that the individual has reached a *tipping point* or threshold. Fourth, Reserve is something that is acquired, built and maintained over a lifetime, beginning with early-life and pre-morbid experiences, and continuing with current experiences and practices, both of which are stimulating to different parts of the brain. The level of activity and the diversity of the domains of stimulation both confer more Reserve.

This more comprehensive way of conceptualizing Reserve invokes a number of suggested directions for Reserve research. We will discuss ‘habits’ in current research on Reserve from the perspective of this broader conceptualization of Reserve. While the limitations associated with these habits are well understood by many in the Reserve field, we believe formally describing these will provide a basis for people less familiar with the field. Then, we will propose nomenclature that uses specific terms embedded in a theoretical model and crosses disciplines, as well as a broader measurement approach. Building on this comprehensive conceptualization, we will discuss potentially fruitful directions for future research.

## Main text

### Changing the habit of measurement: Reserve is more than just education and head size

While it is always appealing to operationalize a complex concept with a simple and easy-to-collect measure, we believe that this approach has substantial drawbacks for research on Reserve, especially when education and head size are used as surrogate markers for Cognitive and Brain Reserve, respectively. Education is one aspect of brain enrichment, but not the whole concept. The use of education as a surrogate limits Reserve research in a number of ways. First, it ignores details related to childhood- and pre-morbid enrichment activities. There is growing evidence that childhood exposure to a broad range of enrichment activities has distinct and notable effects on health and resilience [26]. These include but are not limited to social club participation [27], physical exercise [28], foreign language learning [29], and hobbies [30]. Second, educational attainment measures ignore one’s current activities. These may have a greater impact on health outcomes than past activities [31]. Finally, educational attainment used on its own is confounded by other factors, such as socioeconomic status [32] or “school smart” intelligence [33]. Therefore, although education certainly plays a role in Reserve research, educational attainment alone is insufficient to describe fully a person’s Reserve. Akin to the idea of multiple intelligences [34], Reserve may be better thought of as a multi-dimensional and dynamic concept that is changeable with training.

### Changing the habit of nomenclature: Reserve is more than cognitive

Many of the original studies on Reserve have focused on Cognitive Reserve, but research on Reserve that has considered a set of activities beyond just cognitive exercises has also been fruitful. In particular, research has suggested that relevant enrichment activities extend to a multidimensional array of activities, including physical, cultural, intellectual, communal, and spiritual pursuits [31, 35, 36]. Although all of these pursuits certainly have cognitive components, they are more than cognitive and influence many other domains of life than merely the cognitive domain [31]. Of particular importance, these activities cut across energy-expenditure and content areas and are eminently accessible. Our research has, for example, documented that individuals with MS across the disability spectrum can engage in Reserve-building activities [37] (i.e., physical, cultural, intellectual, communal, spiritual, and lifestyle pursuits), and that higher scores on a patient-reported measure of these activities show consistent and robust associations with health outcomes [31], including lesser symptom burden, lower neurocognitive impact on daily functioning, higher levels of well-being [38]; lesser disability progression over 6 years of follow-up, after covariate adjustment [37] (the emergent nature of the proposed phenomenon of Reserve which will be described more fully below; this addresses the potential tautology that those who do more have more Reserve).

Reserve may also span person characteristics, such as attitudes, values, and socio-emotional skills, rather than only behaviors. For example, cross-sectional data suggest that people with high levels of current Reserve-building activities in their life also tend to think differently about quality of life, characterized by a greater emphasis on the positive, focusing on aspects of their life that are more controllable, and being less based in fantasy [39]. These findings are consistent with research that suggests that if stressors are perceived with a more positive frame they can have a protective effect [40]. Further, people can change how they manage their illness so that they cope better, with a measurable impact on new brain lesion development [41].

These attitudinal differences may reflect personality characteristics. For example, past Reserve-building activities (i.e., educational and occupational attainment; childhood enrichment activities), are positively correlated with the personality trait of openness; and childhood social activities are correlated with extraversion, agreeableness, and conscientiousness [42]. Other relevant person-characteristics include having insight into one’s internal bodily cues (i.e., interoception) [43, 44], having a honed sense of emotional intelligence and/or the ability to read people accurately [44, 45], and valuing perseverance [46]. These skills may develop with age, and can be honed via behavioral or mindfulness intervention [47].

### Proposed nomenclature and model for Reserve

**Fig. 1:**
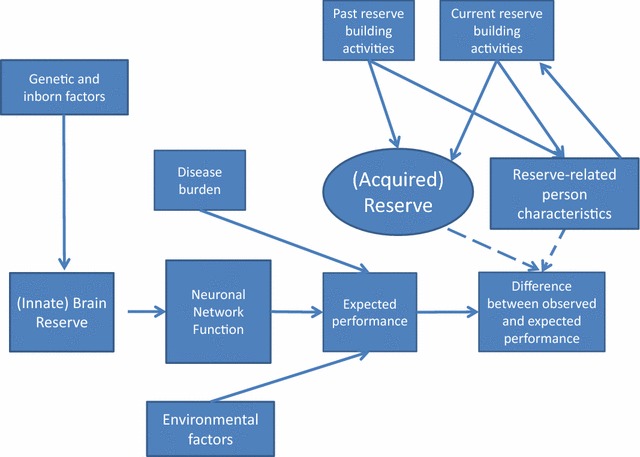
Cross-sectional relationships between components of Reserve and performance. This model provides a roadmap for the nomenclature and expected relationships among Reserve-related constructs at a specific point in time. Going counter-clockwise from left, “*Genetic and inborn factors*” refer to inborn or background determinants of brain function (e.g., single nucleotide polymorphisms). These factors are the only direct causes of (innate) *Brain Reserve*, which represents a subject’s potential brain structure (e.g., head size, intracranial volume, synapse count, Central Nervous System (CNS) structure). Regardless of a subject’s Brain Reserve, the subject’s *Neuronal network function* represents the present level of functioning of a subject (e.g., functional connectivity as measured by functional magnetic resonance imaging). Then the combination of a subject’s present *Neuronal network function, Environmental Factors* (e.g., socioeconomic adversity or advantage; stressful events) and *Disease Burden* (e.g., diagnosis, symptoms, treatment side effects, progressive disability) determine the subject’s *Expected Performance* on a task. Finally, the *Difference between Observed and Expected Performance* is impacted by the person’s *Expected Performance*, (acquired) *Reserve and Reserve-Related Person Characteristics*. *Reserve and Reserve-Related Person Characteristics* are each hypothesized to lead to larger differences between observed and expected performance, but through different mechanisms. Whereas Reserve relates specifically to compensatory or protective brain function, Reserve-related person characteristics refer to attitudes, values, or socio-emotional skills that are posited to enhance an individual’s resilience in the face of adversity and / or disease. Both Reserve and Reserve-related person characteristics are posited to be directly affected by the individual’s *past- and current Reserve-Building Activities*. Such activities are hypothesized to include a multidimensional array of activities that promote brain health, including cultural/intellectual pursuits, physical activity, social/community participation, spiritual/religious practices, and dietary/lifestyle habits

We thus propose the nomenclature and theoretical model shown in Table 1 and Fig. 1, respectively, which expand the breadth and depth of the concept of Reserve by making distinctions among relevant concepts. Going counter-clockwise from left, “Genetic and inborn factors” refer to inborn or background determinants of brain function (e.g., single nucleotide polymorphisms). These factors are the only direct causes of (innate) *Brain Reserve,* which represents a subject’s potential brain structure (e.g., head size, intracranial volume, synapse count, central nervous system (CNS) structure). Regardless of a subject’s Brain Reserve, the subject’s *Neuronal Network Function* represents the present level of functioning of a subject (e.g., functional connectivity as measured by functional magnetic resonance imaging). Then the combination of a subject’s present *Neuronal Network Function*, *Environmental Factors* (e.g., socioeconomic adversity or advantage; stressful events) and *Disease Burden* (e.g., diagnosis, symptoms, treatment side effects, progressive disability) determine the subject’s *Expected Performance* on a task. Finally, the *Difference between Observed and Expected Performance* is impacted by the person’s *Expected Performance*, (acquired) *Reserve* and *Reserve*-*Related Person Characteristics*. *Reserve* and *Reserve*-*Related Person Characteristics* are each hypothesized to lead to larger differences between observed and expected performance, but through different mechanisms. Whereas Reserve relates specifically to compensatory or protective brain function, Reserve-related person characteristics refer to attitudes, values, or socio-emotional skills that are posited to enhance an individual’s resilience in the face of adversity and/or disease. Both Reserve and Reserve-related person characteristics are posited to be directly affected by the individual’s *past*- *and current Reserve*-*Building Activities*. Such activities are hypothesized to include a multidimensional array of activities that promote brain health, including cultural/intellectual pursuits, physical activity, social/community participation, spiritual/religious practices, and dietary/lifestyle habits.

**Table 1:** Reserve-related constructs and suggested operationalization

Construct	Definition	Potential measures
Genetic and inborn factors	Background determinants of brain function	Single nucleotide polymorphisms
Brain Reserve	Brain structure	Head size, intracranial volume, synapse count, structural magnetic resonance imaging
Neuronal network function	Made up of the brain, spinal cord and nerves, the central nervous system (CNS) is responsible for integrating sensory information and responding accordingly	Functional magnetic resonance imaging
Environmental factors	Contextual factors specific to the person that may constrain or facilitate functioning	Stressful events (e.g., job loss, death of a loved one) or socioeconomic adversity (e.g., inability to pay bills, unsafe neighborhood, social isolation, etc.) or advantage (e.g., financial security, safe neighborhood, community connection, opportunity)
Disease burden	Assaults to the brain due to disease or injury	Structural magnetic resonance imaging (e.g., lesion load, atrophy)
Reserve	Compensatory or protective factor that limits the impact of assaults to the brain from the disease or injury. When low, then impact of assaults to the brain are magnified	The impact of Reserve on CNS functioning can be inferred by estimating the impact of past and current-Reserve building activities because the path from the activities to CNS functioning is through Reserve
Reserve-building activities	Past and current achievement (occupational, educational) as well as enrichment activities across a range of domains (physical, cultural, intellectual, communal, spiritual, and lifestyle pursuits)	Patient-reported outcome measure such as the DeltaQuest Reserve-Building Activities Measure©
Reserve-related person characteristics	Attitudes, values, and socio-emotional skills	Person-reported measures of perseverance, work value, and socio-emotional intelligence resources. May also consider measures of appraisal processes and personality
Difference between observed and expected performance	Difference been observable performance on a task and the performance expected based on available covariates	Performance-based metrics such as cognitive, motor, and behavioral measures reflecting neurocognitive processing speed, executive function, physical functioning, emotional health, and/or disability

In the proposed conceptualization, “Reserve” is an emergent phenomenon or latent construct (as shown by being in an oval rather a rectangle in Fig. 1) that is inferred when there is a discrepancy between expected and observed functioning. For example, there are people who perform better (i.e., better neurocognitive processing speed or self-reported physical, social or emotional functioning) than would be (normatively) expected given their observed neurological deficits (e.g., brain lesion volume in the case of MS). There would be no basis for considering Reserve without such discrepancy. Reserve would be estimated using a residual modeling approach similar to that suggested by Reed et al. [48], where it is defined as the explained variance in the impairment-performance discrepancy, after adjusting for covariates reflecting other boxes in the Fig. 1 model.

We further propose thinking about Reserve and Reserve-building activities in terms of Brunswik’s lens model [49]. In other words, many inputs come into focus to build Reserve which in turn radiates to many self-management challenges and many outcomes. It is likely that it is not only the level of engagement that matters, but also the breadth of activities in which the individual is involved. This idea is consistent with Lawton and Nahemow’s *Competence Press Hypothesis* [50], which says that individuals increase their adaptive capacity when they are called upon to meet environmental demands that they find challenging but not overwhelming. We would hypothesize that breadth and level would be associated with greater Reserve. Some of these activities may be historical (past Reserve-building). Some activities may be explicitly intentional to build brain function, whereas others may be incidental.

We argue that Reserve is a common pathway—that results from the brain-strengthening aspects of Reserve-building activity. Evidence of Reserve ought to be manifest very specifically in improved self-management of disease. In other words, there is no specific relationship between any given type of Reserve-building to any given performance challenge; the benefits accrue in a variety of ways and benefits may be realized in many different ways.

Since Reserve is directly impacted by past and current *Reserve*-*Building Activities,* Reserve and Reserve-related person characteristics are considered modifiable. It is also hypothesized that Reserve is multidimensional, including cognitive, physical, socio-emotional, and spiritual. Depending on the nature of the research question and the domain(s) of interest, one might focus on one or more domain. From a statistical analysis perspective, the impact of Reserve on the difference between observed and expected performance can be inferred by estimating the impact of past and current-Reserve building activities because the path from the activities to the difference in performance is through Reserve. This approach has been used in the research showing that greater education reduces the impact of reduced brain volume on cognitive performance in MS patients [19], but our model provides theoretical grounding for this analysis.

Because Reserve building activities are choose-able and therefore modifiable, these activities are relevant and exciting from a public health perspective. Individuals can engage in day-to-day activities that build resilience. Based on our and others’ research, we believe that Reserve-building activities and person characteristics contribute to better control of disease processes and self-care. Reserve-building is *not* merely skill-building, like helping people plan meals or adhere to medications. Rather, it improves outcomes broadly and indirectly, by enhancing general executive function and general resilience as well as the motivation to self-care.

It is important to note that we have moved away from the terms “Cognitive Reserve”, and “Neurological Reserve” that have been commonly used in the literature, but the term “Brain Reserve” has been retained to have the same meaning as in previous work [7]. Many past research studies have focused on *Cognitive* Reserve—the relationship between cognitive performance, brain function, and disability (e.g., [51]). Others focus on *Neurological* Reserve, emphasizing the relationship between brain structure and neurological disability (e.g., [52]). We have chosen to focus on a broader multidimensional concept of “Reserve”, the activities that are hypothesized to strengthen it, and the person-characteristics relevant to its expression. We have also distinguished between inborn/unchangeable aspects of Reserve (Brain Reserve), and what are hypothesized to be modifiable aspects of Reserve (subsequent to past and current Reserve building activities). All of the above dimensions of Reserve—cognitive, physical, socio-emotional, and spiritual—are relevant to individuals’ health, well-being, and disease trajectory, and thus should be considered in future observational and intervention research.

### Changing the habit of intra-disciplinary thinking: reconsidering disease trajectory through the Reserve lens

**Fig. 2:**
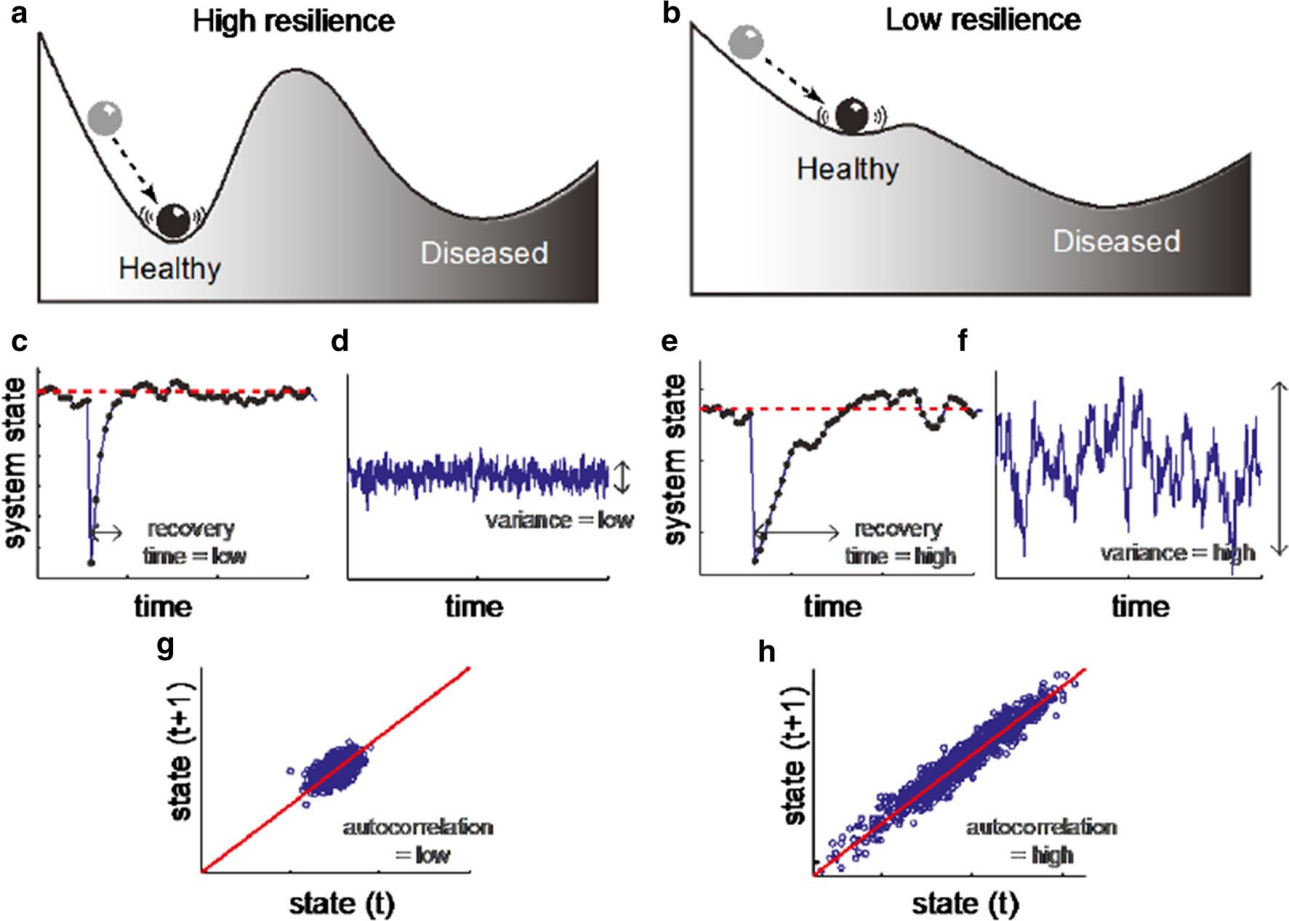
Critical characteristics reflecting distance from tipping point. Loss of Reserve can be identified by early warning signs (i.e., slope, time to recovery, variance, and autocorrelation), and may explain different phenotypes of a disease (reprinted with permission from Wolters Kluwer from Olde Rikkert [54: 605])

Interdisciplinary thinking can lead to paradigm shifts that are critical for solving what had been considered unsolvable. As an example, the concept of a “tipping point” originated in studies by Scheffer and colleagues to understand lake ecosystems that changed from healthy states to unhealthy states [53]. His characterization of this shift identified specific and recognizable phenomena that preceded the changes—‘early warning signs’. This mathematical model has relevance in predicting shifts from high- to low-functioning across conditions as diverse as epilepsy, asthma, heart and renal failure, migraine, and cardiac arrhythmias [54]. Figure 2 illustrates key characteristics:

- The concept of a tipping point is illustrated in panels a and p, where the patient is represented by the ball and that landscape represents the patient’s relative stability or instability with regard to health transitions. It would take considerably more of a push to move the first patient (a) than the second patient (b) to a state of poor health. Thus patient b is at or past the tipping point, whereas patient a is far from the tipping point. The more steep slope in the high versus low resilience panels (a vs. b), suggests lower ‘difficulty’ in switching in the low-resilience condition;
- The characteristic of a slowing down of recovery upon perturbations is illustrated in panels c versus e. In panel e, it takes longer to return to equilibrium.
- The characteristic of increased variance in randomly-induced fluctuations is shown in panels d versus f. Small external stressors affect the current state of the person more in panel f than in panel d, illustrating a smaller buffer against small external stressors.
- The characteristic of increased ‘memory’ or autocorrelation between the serial measurements is indicative of lower resilience. As the disease state moves toward its tipping point (g vs. h), there are increases in systematic variation due to decline or recovery. Thus time *t* + 1 is likely to be closer to *t*, reflecting a higher autocorrelation.

Considering Reserve in the context of tipping point phenomena would suggest that when a subject has accumulated a sufficient amount of disease burden and/or environmental effects, Reserve would be unable to fully overcome these detrimental effects. Alternatively, if Reserve is depleted, a subject’s observed performance would equal the expected performance. This is also consistent with the concept of Brain Reserve as a fixed threshold/passive process [55] where after a fixed cut-off or “point of inflection” functional impairment is assumed to occur for everyone [9, 56]. This inability of Reserve to overcome the detrimental effects would be inferred, for example, if the individual’s observed performance showed occasional and then more frequent declines. Early warning signs might be a slowing down or increased variance on some disease-specific metric of performance (e.g., timed 25-foot walk, cognitive processing speed), change in physical activity (e.g., exercise is the first thing to go when one is stressed), or in health outcomes such as affective state (e.g., mood fluctuations), or vitality (e.g., fatigue). We postulate that if Reserve is insufficient, patients can experience irreversible changes in health state.

It is possible that this phenomenon occurs in multiple diseases including MS, Alzheimer’s disease, Parkinson’s disease, diabetes, and chemotherapy-induced functional problems. It is known, for example, that people who are more physically fit are able to withstand treatment toxicities better than those with lower fitness levels [57]. Further, it is known that physically fit individuals exhibit a *compression of morbidity* at the end of life, that is that they live as long or slightly longer than the average but experience morbidity for a shorter time at the end of life, rather than a progressively disabled trajectory over a period of years [58]. Our Reserve theory would predict that people engaging in more Reserve-building activities—not just physical exercise—would presumably increase Reserve and consequently lead to a compression of morbidity at the end of life.

Thinking in terms of a tipping point implies focusing on inter- and intra-individual variability may anticipate onset of worsening disease course. Tipping point adds to the existing concept of point of inflection [8, 58] by providing methods for estimating an individual’s ability to maintain homeostasis in functioning in response to stressor-event triggers and health status [59]. Building on these tipping point models can thus help to identify efficiently where an individual is in the high-low resilience continuum, and may improve critical care and acute disease management. Methods borrowed from the field of ecological momentary assessment [60] can be applied to examining temporal patterns of Reserve-building activity, and seeing how they relate to stability and variation in capacity and performance.

## Conclusions

### Implications for future research

Reserve research has great potential to improve health and well-being across many disease groups as well as in healthy populations. It has direct applications across the age continuum, suggesting that interventions to improve educational programs for children and adults of all ages can benefit from engaging in a growing and changing breadth and depth of exposure to physical, intellectual, cultural, social, and spiritual activities. Reserve is an empowering concept because it suggests that one can have a palpable impact on preventing ill health and disability progression by engaging in a varied repertoire of enriching activities. We suspect that it is not one specific activity or set of activities that matters, but rather the engagement and stimulation invoked by such practice. Documenting the importance of such broad exposure to stimulating activities, particularly in the context of leisure activities, may have important policy implications.

The proposed model is a starting point for future research, and its limitations should be acknowledged. First, in the interest of simplicity of presentation, our figure shows relationships between constructs with single arrows. It is, however, likely, that interaction among variables (i.e., moderation effects) exist that are not shown in the model (e.g., between acquired Reserve and person characteristics). It is also possible that there are mediation effects among variables in the model. Future research should operationalize the constructs related to Reserve in ways shown in Table 1, and examine possible moderation and mediation effects among the measured constructs. This measurement approach retains the distinction among the constructs, and allows diverse measurement modes to triangulate on characterizing these related constructs. Prospective work using advanced neuroimaging techniques evaluating (e.g., using fMRI to study brain plasticity) is needed to test the causal relationship between Reserve-building activities and disease progression. It would important to know, for example, whether people who maintain various Reserve-building activities over time show a slower rate of progression of symptom burden. Do they persist in a stable way that buffers the individual against early warning signs of a tipping point?

Future research should also utilize design strata that will allow one to separating an individual’s socioeconomic status (e.g., income) and intelligence from the broader concept of Reserve. Does Reserve have a similar relevance to special needs populations? To people who have ‘normal’ intelligence? To people who do not score well on standardized academic metrics of intelligence but excel in other aspects of (multiple) intelligence, such as artistic ability, spatial skills, construction, etc.? How does Reserve differ among ‘workaholics’, that is people who spend most of their waking hours working and thus almost never engage in stimulating activities outside of work? Relatedly, future work should focus on the unique challenges of measuring Reserve in childhood or adolescence, as well as the potentials of the new proposed conceptualization in this young population. Given the active (re)organization and growth of the brain during childhood and adolescence, it is likely increasingly important to ensure the inclusion of Reserve-building activities in one’s lifestyle. Future research might evaluate whether interventions to increase the level and frequency of Reserve-buiilding activities in children or adolescents with MS, for example, has an impact on long-term disability progression. Finally, how does Reserve relate to emotional intelligence and social connection? All of these questions have yet to be studied, and would have important implications for interventions to improve health and well-being for people with health concerns. It is our hope that this discussion of Reserve will encourage and enable more comprehensive research and applications of this important concept.

## Abbreviations

CNS: central nervous system
MS: multiple sclerosis
